# On the concept and elucidation of endogenous retroviruses

**DOI:** 10.1098/rstb.2012.0494

**Published:** 2013-09-19

**Authors:** Robin A. Weiss

**Affiliations:** Division of Infection and Immunity, Wohl Virion Centre, University College London, Cruciform Building, Gower Street, London WC1 6BT, UK

**Keywords:** retrovirus, Mendelian inheritance, germ-line infection, receptors, tropism

## Abstract

Endogenous retrovirus (ERV) genomes integrated into the chromosomal DNA of the host were first detected in chickens and mice as Mendelian determinants of Gag and Env proteins and of the release of infectious virus particles. The presence of ERV was confirmed by DNA hybridization. With complete host genomes available for analysis, we can now see the great extent of viral invasion into the genomes of numerous vertebrate species, including humans. ERVs are found at many loci in host DNA and also in the genomes of large DNA viruses, such as herpesviruses and poxviruses. The evolution of xenotropism and cross-species infection is discussed in the light of the dynamic relationship between exogenous and endogenous retroviruses.

## 1. Introduction

Nowadays, the notion that viral genetic sequences are present in host genomes is commonplace [1,2]. Conversely, many host genes have been incorporated into large DNA viruses, such as herpesviuses and poxviruses, as well as oncogene-bearing retroviruses. In the late 1960s, however, when Jim Payne and I first saw evidence, respectively, of Gag and Env proteins expressed by uninfected chickens, it seemed too outlandish to take seriously. Reverse transcriptase had not yet been discovered, although Howard Temin had recently enunciated his DNA provirus hypothesis [3]. He postulated that RNA tumour viruses made DNA copies which then integrated into host chromosomal DNA, analogous to integration of prophage in bacteria. The idea was viewed with considerable scepticism until the discovery of reverse transcriptase in 1970. The DNA provirus hypothesis involving reverse transcription and integration is generally regarded as a revolutionary paradigm shift, but the science historian Fisher suggests in a recent reappraisal that Temin was actually thinking—albeit boldly—within the conceptual framework of his time and that the synthesis of DNA from an RNA template did not overturn the ‘central dogma’ of molecular biology [4]. If the RNA world came before the evolution of DNA and proteins, one could argue that reverse transcription is actually a fossil relic turned to good use alongside telomerase and other oddities of nature [5].

I first came across an endogenous factor which functionally complemented *env*-defective Rous sarcoma virus (RSV) during my doctoral studies in 1966. That was the year that Peyton Rous was awarded the Nobel Prize for Medicine or Physiology for the isolation of his eponymous virus published in 1911, representing a record 55 year incubation period between reporting a discovery and the award [6]. The recognition of the importance of Rous's discovery after such a long delay was largely owing to the cell transformation assay in monolayer culture of chick embryo fibroblasts reported by Temin & Rubin [7] in 1958 which enabled quantitative experimental studies of virus replication and cell transformation.

Integration of viral DNA into host DNA was first discerned for the prophage of the temperate bacteriophage lambda by Andre Lwoff in 1950 and for the simian DNA virus SV40 in cultured mammalian cells in 1968 [8]. For small DNA tumour viruses, the full replication cycle occurs via non-integrated circular viral genomes, whereas viral integration into host DNA usually leads to abortive infection and sometimes to cell transformation. In contrast, and in full confirmation of Temin's DNA provirus hypothesis, retroviral integration is an obligatory step in replication, whereas non-integrated 2-LTR circles lead to abortive infection. The idea that retroviral infections would occur in the germ-line and become inherited through countless generations by the host required a further leap in imagination. Indeed, germ-line integration has not yet been described for DNA tumour viruses, although we now know that it occasionally occurs with human herpesvirus 6 [9,10].

## 2. How endogenous retroviruses came to light

I have previously described how endogenous retroviruses (ERV) were discovered [11], so a summary of the evidence will suffice here. Endogenous genomes were first found in avian alpha-retroviruses, and soon after in murine beta- and gamma-retroviruses. These are ‘simple’ retroviruses lacking auxiliary genes to *gag*, *pol* and *env*. More recent evidence shows that the ‘complex’ spumaviruses and lentiviruses also have endogenous representatives in host species ranging from sloths to rabbits [12–14]. There is little evidence yet of endogenous versions of delta-retroviruses related to human T-lymphotropic virus type 1 and bovine leukosis virus.

The evidence for ERV was accrued first through virological and immunological observations, followed by molecular biological evidence of viral DNA hybridization to host DNA. The replacement of liquid DNA hybridization by Southern blotting revealed multiple endogenous insertions at many chromosomal loci. Since complete sequencing of host genomes was introduced, data mining and bio-informatic searches show how much DNA is of retroviral origin, ranging from single LTR inserts to complete genomes. For example, approximately 8% of human DNA represents fossil retroviral germ-line insertions [15].

### (a) Avian endogenous retroviruses

In chickens, an immunological assay for Gag antigen was used to screen flocks to determine whether they harboured the alpha-retrovirus, avian leukosis virus (ALV). However, the specificity of this test was confounded by the presence of a cross-reacting antigen in some birds. This Gag-like antigen was shown to be inherited as a simple dominant allele in certain breeds of fowl [16]. Meanwhile, the *env*-defective Bryan strain of RSV was observed to release infectious virus from certain chick cells [17,18]. This observation was attributed to a cellular Env glycoprotein with novel largely xenotropic host range infecting quail and pheasant cells, and a distinct neutralization phenotype [18–20]. The *env* and *gag* markers were then shown to be inherited as a single host locus [21]. In addition, another breed of fowl spontaneously released an infectious leukosis virus, RAV-0, with the same envelope properties as the complemented RSV [22]. The release of a similar virus could be induced by treatment of embryonic fibroblasts with mutagens [23].

With the introduction of DNA hybridization methods, similar amounts of ALV DNA were found to be present in chicken DNA from phenotypically negative breeds as well as positive breeds [24]. Later, it became apparent by Southern blotting that numerous separately integrated endogenous proviruses are present in the chicken genome [25]. Some ERV genomes were full length with open-reading frames representing potentially replication-competent viruses, while others were defective or not expressed at all. Analysis of the four extant species of jungle fowl showed that only red jungle fowl (the main ancestor of the domestic fowl) carried ERV sequences homologous to ALV. This finding indicated that the alpha-retrovirus endogenous sequences had colonized the germ-line very recently in evolution, before domestication but after speciation of the genus *Gallus* [26]. The small numbers and insertional polymorphism of endogenous ALVs permits them to be bred out of chickens, with no harmful consequences. Today, using the complete genomes known for three species of birds, numerous types of endogenous alpha-, beta-, alpha–beta-intermediate and gamma-retroviral genomes have been discerned in birds [27].

### (b) Murine mammary tumour virus

Murine mammary tumour virus (MMTV) is the prototype beta-retrovirus. Breast cancer susceptibility in mice has taken a full circle in being viewed first as a genetic disease, then as an infection and then back to inheritance as a Mendelian provirus in certain mouse strains. In the early twentieth century, different inbred strains of mice were selected for having either a high incidence (C3H) or a low incidence (C57Bl) of breast cancer. In 1936, Bittner demonstrated that the incidence of breast cancer was determined by a transmissible factor in the milk so that a low strain mouse would develop breast cancer if suckled on a high strain foster-mother, and vice versa [28]. The milk factor is MMTV and thus the vertical transmission did not appear to be genetic after all but due to retroviral infection. However, shortly before the announcement of the discovery of reverse transcriptase, Peter Bentvelzen and co-workers [29,30] showed that in the GR strain of mouse, MMTV and associated high mammary carcinoma is inherited as a Mendelian trait. Again, there are multiple copies of MMTV in the murine genome [31], some of which encode super-antigen functions that induce non-specific T-cell activation [32]. Multiple reinsertion into the somatic murine genome and activation of adjacent oncogenes such as those of the *wnt* and *fgf* families result in mammary carcinogenesis [33].

### (c) Endogenous gamma-retroviruses in mammals

Murine leukemia virus (MLV) is the protype gamma-retrovirus. There was evidence throughout the 1960s that latent MLV could be activated by radiation of apparently uninfected mice and that host genetics played an important role in MLV control and expression, but germ-line transmission of MLV was not conceptualized at that time. The Akr strain of inbred mice has a high incidence of thymic lymphoma associated with MLV, and it became a favoured model for viral leukaemogenesis. The true endogenous nature of the provirus was revealed when virus was induced in uninfected embryonic fibroblast cultures by treatment with bromodeoxyuridine [34,35], which releases gene silencing caused by DNA methylation.

In the genome of Akr mice, replication-competent MLV is integrated at two loci, *Emv*-11 and -12. They are examples of ‘ecotropic’ viruses, which can replicate to high titre in the species in which they are inherited; others are ‘xenotropic’ as they can infect human and other mammalian cells but not reinfect cells of other common inbred mouse strains, and others are ‘polytropic’ as they can infect a broader range of hosts. Jay Levy coined these terms of tropism and host range when he first discovered xenotropic virus in New Zealand black/white hybrid mice [36]. Further endogenous MLV are polytropic in being able to infect several host species, and recombinant infectious retroviruses can result from the activation of these genomes [37]. The host range of these MLV strains is mainly determined by the envelope glycoproteins interacting with different receptors.

During the 1970s, ERVs were found in numerous other mammalian species. For instance, infectious viruses are produced in the placenta of baboons [38], which represents a recombinant gamma-retrovirus with a beta-*env* [39]. A related ERV called RD114 was found in a human tumour cell line RD, which had been xenografted through a fetal kitten brain. RD114 was reported to be a human gamma-retrovirus when it was first described in 1972, although it was really an endogenous feline retrovirus; this and other evidence of retroviral tissue contamination [40] give a feeling of déjà vu to the recent XMRV episode where an association with disease turned out to be the result of contamination with murine retroviruses.

## 3. Genetic recombination between exogenous and endogenous retroviruses and pathogenesis

Endogenous retroviral genomes were originally derived from exogenous infections of the germ-line. While some integration sites are ancient, others arise through activation and reinfection, as well as entry of new infectious viruses into a species throughout evolution. This has been mapped in the primate lineage leading to humans, where successive waves of HERV-K genomes have recolonized the host DNA [41]. When activated to express RNA genomes with packaging sequences and packaged into infectious virions, ERV can recombine among themselves or with exogenous retroviruses.

Recombination between exogenous and endogenous retroviruses was first demonstrated using a non-defective strain of exogenous RSV with a host range marker determined by *env*. A high-frequency genetic recombination occurs with endogenous *env*, provided that it is expressed and packaged into retroviral particles [42]. Recombination between gamma-retroviruses is an important feature of leukaemogenesis. In mice, recombination between endogenous ecotropic and xenotropic LTR sequences and with polytropic *env* sequences occurs to generate a virus, which uses the polytopic receptor [37]. The high virus load and replicative capacity of the recombinant virus leads to multiple new integration sites associated with thymic lymphomas. Thus, a complex pattern of recombination as well as insertional mutagenesis eventually leads to disease [43].

In cats, the exogenous feline leukaemia virus (FeLV) with an A envelope recombines with endogenous *env*s to give rise to leukaemogenic FeLV-B [43,44]. On the other hand, the emergence of FeLV-C, which is associated with anaemia, occurs through hypermutation of exogenous FeLV-A *env* sequences [45]. In both the cases, the variant, pathogenic strains arise anew in each affected cat; only FeLV-A appears to be readily transmissible in the feline population, although FeLV-B and FeLV-C are infectious *in vitro* [46].

The recombinant retroviruses that encode oncogenes are seldom, if ever, naturally transmitted. Most *onc*-bearing retroviruses are replication defective but can be experimentally transmitted *in vitro* or by *in vivo* inoculation when replication-competent helper virus is present in excess to provide the missing replicative or structural proteins [6]. It is noteworthy that the numerous oncogenes found in retroviruses have only been discovered because experimental pathologists such as Rous have sought to identify and transmit them.

## 4. Evolution of xenotropism and cross-species infection

Generally speaking, endogenous retroviruses, which remain at their inherited loci, do not cause malignant disease until they become activated and undergo multiple rounds of replication in somatic cells. The generation of such numerous integration events by newly synthesized proviruses results in rare insertional activation of cellular oncogenes due to the regulatory signals contained within the LTRs [43]. It follows that it may be of selective advantage to the host to restrict the *in vivo* spread of retroviruses [47]. Restriction factors other than receptors are discussed by other contributors to this theme issue. Here, I briefly comment on receptors because they were an early example of host restriction in the study of ERV.

I view xenotropism as an example of the host evolving restriction to a retrovirus by mutating its receptor. The non-functional receptor prevents further spread between hosts and also restricts an activated endogenous retrovirus propagating to high virus load and multiple new integration sites. Certain mutations in cell surface receptors which no longer allow endogenous retroviruses to bind or enter cells would not disable the normal, physiological functions of the receptors [48]. These would therefore be of selective advantage to the host. However, the ‘Red Queen’ arms race and resulting counter-evolution by the virus would give rise to variants that could adopt alternative receptors, e.g. polytropic MLV [37].

In hosts which have not been exposed to or invaded by a particular retrovirus, receptor mutations will not be subject to selection. Hence the virus will appear to be xenotropic in that it can potentially infect cells of naive species. It is interesting that exogenous, infectious gamma-retroviruses have not been found in humans, which is likely due to restrictions other than receptors in primary cells [47]. Xenotropic MLV and feline RD114 grow well in many established cell lines of human origin, which do not express restriction factors such as APOBEC, TRIM5 or tetherin.

It is remarkable how certain ERV have jumped by infection to colonize phylogenetically distant hosts. ERV of South East Asian species of mice (*Mus caroli* and *M. cervicolor*) [49] are closely related in genome sequence and presumably are ancestral to the exogenous retrovirus found in captive gibbon [50], and also in marsupial koalas where it is becoming endogenous in the germ-line [51]. Similarly, the feline ERV, RD114, resembles an ERV genome, which is widespread in various species of baboon and gelada. RD114 occurs in tabby species (but not spotted species) of *Felis* such as the Egyptian sand cat (probably the ancestor of the domestic cat) and the European wild cat [52]. It seems clear that the source of the original infection was from primate to feline and not the other way round. The mechanism of transmission remains speculative; however, since the baboon virus is highly expressed in the placenta, one can envisage scavenging cats feeding on primate afterbirths. Perhaps, cats also picked up their MLV-related FeLV through preying on mice. Although most ancient ERV genomes are defective, infectious recombinants can emerge from them [53]. These examples of cross-species infection illustrate how long-term ERV residents in one species can colonize new unrelated species, cause epidemics and eventually become ERV in their newly adopted host.

## 5. Endogenous retroviruses that are useful to their hosts

ERV can be put to physiological use by their hosts, either at the gene regulatory level or as proteins. Tissue-specific enhancers in the ERV LTR are an example of transcriptional control. All mammals express amylase in the pancreas, but rodents and Old World primates also express amylase in salivary glands. In both the cases, ERV elements play a role in salivary expression in remarkably convergent evolution. The activation of salivary amylase in the human parotid gland is controlled by a retroviral insertion, which occurred during Old World primate evolution [54]. Then gene triplication of the amylase gene and its LTR enhancer to further enhance amylase secretion occurred after hominids split from chimpanzees. It may have provided selective advantage to the hominid lineage when, like rodents, they adopted a diet containing complex carbohydrates.

Many years ago, it was noted that MLV is not transcriptionally active in murine embryonal carcinoma stem cells, but that during differentiation into somatic cells MLV genes begin to be expressed [55]. This suppression is now largely explained by the restriction factor TRIM28 binding to the MLV LTR via the zinc finger protein, ZFP809 [56]. In contrast, recent evidence indicates that HERV-H is highly expressed in human embryonic stem cells and in reprogrammed cells as a precise marker of pluripotency, but it becomes silenced on differentiation into embryoid bodies [57]. It is not yet understood whether the stem cell silencing of endogenous MLV and activation of HERV-H are simply correlative markers or whether they play a functional role in development, but my guess is the latter.

The advantage to the host of ERV protein expression is most dramatic in the evolution of the mammalian placenta. We suggested that the ERV-3 envelope might function in placental trophoblast cell fusion and immunological protection of the fetus [58], but it later became apparent that this HERV is not universally present in human genomes and therefore cannot be essential for placental function. However, the idea, if not the particular HERV, turned out to be correct [59]. In various orders of placental mammals, the fusogenic properties of different endogenous retroviral glycoproteins called syncytins have been turned to physiological benefit for the formation of the syncytiotrophoblast [60].

ERV glycoproteins might also be useful in blocking receptors against exogenous retrovirus infection or against activated replicating ERV. This phenomenon used to be termed retroviral interference but is now usually called super-infection resistance [61]. Cells that are productively infected by retroviruses are resistant to super-infection with strains using the same type of receptor, because these receptors are already occupied or down-modulated from the cell surface. The subtypes of avian and FeLVs were originally classified on this basis and super-infection resistance was used to reveal seven distinct human receptors for beta- and gamma-retroviruses [62].

If an ERV expresses Env, even in the absence of whole virus particles, the endogenous glycoprotein can block the receptor. Super-infection resistance by ERV was first observed with the endogenous ALV glycoprotein [63] and the same mechanism of action explains the *Fv-4* restriction factor for ecotropic MLV [47,64]. The non-replicative expression of endogenous Env might thus be advantageous to the host in blocking infection or reducing viral load of an exogenous or activated endogenous replication-competent virus. It may therefore be a driver for the endogenization of recently acquired exogenous retroviruses. The transmissible koala retrovirus associated with leukaemia could be a case in point [51]. By becoming an endogenous genome it might protect koalas from infection by its exogenous, potentially pathogenic precursor.

## 6. Endogenous retroviruses in the genomes of DNA viruses

Retroviruses are typically not particularly choosy about sites of proviral integration while generally preferring active chromatin, although some such as ALV have distinct target site preferences. Therefore, it is not too surprising that retroviral genomes have been detected in the genomes of large DNA viruses. The first example was of sequences of avian reticulendotheliosis virus (REV) in the genome of the avian alpha-herpesvirus, Marek's disease virus [65] and the related turkey virus. REV has also been found in the genome of fowlpox virus [66], a virus which has a purely cytoplasmic replication cycle. It is curious that ALVs have not travelled in the same direction, and that we still lack examples of retrovirus integration into mammalian DNA viruses. Although an avian virus, REV itself appears to have a mammalian origin [67].

## 7. Endogenization of other viruses

Human herpesvirus 6 (HHV-6) is transmitted as a Mendelian element in certain human families [9,10]. However, it is not clear that such sequences are retained in the host genome indefinitely like ERV genomes. It will be interesting to investigate this phenomenon further. Is there any special property to single out HHV-6 from other human herpesviruses for endogenization? Do other vertebrate species contain endogenous herpesvirus genomes or genome fragments?

The presence of cDNA of non-retroviral RNA viruses is discussed in this theme issue. It was first reported for measles virus by Zhdanov in 1975 in Moscow [68] and was greeted as sceptically as the discovery of endogenous retroviruses. Other examples include the non-germ-line accumulation of cDNA of the arenavirus, lymphocytic choriomeningitis virus (LCMV) in host DNA in laboratory-infected murine cells [69]. Bornavirus and filovirus cDNA sequences occur in several mammalian species, including humans [1,70,71]. The bornavirus and filovirus sequences appear to be genuinely endogenous in germ-line DNA as ancient integrants.

These RNA viruses do not, of course, possess reverse transcriptase. One effector agent appears to be the reverse transcriptase of LINE elements and the DNA fragments of RNA viruses may be regarded as pseudogenes. However, the reverse transcriptase of endogenous intracisternal A particles (IAP) may also play a role as LCMV, and bornavirus sequences are found within the IAP provirus [69]. It is intriguing that IAPs and other ERVs that have lost their *env* sequences appear to accumulate more rapidly in greater abundance as endogenous genomes [72] because, unusually for retroviruses, they short-cut extracellular budding and reinfection.

Overall, these examples show how a wide variety of viruses can invade host genomes upon which host natural selection can then exert its effects.
